# The association of diabetes status and bone mineral density among US adults: evidence from NHANES 2005–2018

**DOI:** 10.1186/s12902-023-01266-w

**Published:** 2023-02-01

**Authors:** Bo Liu, Jingshuang Liu, Junpeng Pan, Chengliang Zhao, Zhijie Wang, Qiang Zhang

**Affiliations:** 1 Department of Orthopaedics, Beijing Ditan Hospital, Capital Medical University, Beijing, 100015 China; 2 Department of Emergency Internal Medicine, the Affiliated Hospital of Qingdao University, Qingdao, 266000 Shandong China; 3 Department of Spinal Surgery, the Affiliated Hospital of Qingdao University, Qingdao, 266071 China

**Keywords:** Pre-diabetas, Diabetes, Mineral density, NHANES, Association

## Abstract

**Backgrounds:**

We aimed to explore the relationship between diabetes status and bone mineral density (BMD) among adults with pre-diabetes and diabetes.

**Methods:**

We collected and analyzed five cycles (2005–2006, 2007–2008, 2009–2010, 2013–2014, and 2017–2018) data from NHANES. We removed the individuals containing missing values. The linear regression models were used to explore the relationship between diabetes status and bone mineral density. Finally, we performed subgroup analyzes by age, sex and race to find special populations.

**Result:**

Finally, 9661 participants with complete data were involved in the study. 944 were diagnosed with pre-diabetes, and 2043 were with diabetes. We found that bone mineral density in the hip, femoral neck, and lumbar spine showed an upward trend in both prediabetic and diabetic patients in the three linear regression models. Further, after subgroup analysis, we found that this trend was more prominent in whites race, women, and those over 50 years old.

**Conclusion:**

Using NHANES data from 2005 to 2018, we found that patients with abnormal glucose metabolism had increased bone mineral density.

## Introduction

Diabetes is characterized by hyperglycemia caused by defects in insulin secretion, insulin action, or both. The prevalence of abnormal glucose metabolism is increasing yearly, and the total number has risen from 400 million in 2014 to 470 million by 2030, There was a similar increase in the prevalence of prediabetes [1]. Typically, individuals with prediabetes have blood sugar levels that are slightly elevated, but do not meet the diagnostic criteria for diabetes. Prediabetes can be diagnosed using blood sugar tests, including the fasting blood sugar test (FBS), the oral glucose tolerance test (OGTT), and the random blood sugar test (RBS) [2]. Prediabetes can progress to diabetes, so it is important to take appropriate preventive measures.Diabetes is associated with many complications, including neuropathy, retinopathy, nephropathy, osteoporosis, and cardiovascular and cerebrovascular diseases. In particular, bone strength and bone mineral density were also impaired [3, 4].

Osteoporosis is the most common bone disease and the primary disease affecting the health of 200 million people worldwide, It is a disease of the skeletal system characterized by decreased bone strength, which increases the risk of fractures in the hip, spine and other skeletal sites [5, 6]. Bone mineral density is one of the most commonly used indicators for clinical diagnosis of osteoporosis. In addition to bone density, bone quality is also an important indicator for measuring osteoporosis. However, the detection of bone quality is more complicated, so bone density is more commonly used in clinical practice. If the bone mineral density does not meet the diagnostic criteria for osteoporosis, but there are fragility fractures in related parts, it can also be diagnosed as osteoporosis. Therefore, bone mineral density is one of the commonly used indicators for diagnosing osteoporosis, but it is not the ‘Gold Standard’ .

Many studies have shown that diabetes has an impact on bone metabolism. It can lead to metabolism disorders of sugar, protein, and fat, as well as negative balance of calcium metabolism and abnormal bone metabolism [7, 8]. Diabetes increases the risk of osteoporosis. Similarly, it also increases the risk of hip fracture [9]. However, many studies have also confirmed that diabetic patients have increased bone mineral density [10–12]. It was contradictory that their bone mineral density increased, but the risk of fractures also increased. Therefore, there is still no consistent conclusion on the impact of diabetes on bone mass and bone density. In addition, there are few studies by researchers on the relationship between prediabetes and bone mineral density. Our study collected and analyzed data from the NHANES database from 2005 to 2018. We divided all participants into normoglycemia, pre-diabetic, and diabetic. Our purpose is to clarify the relationship between diabetes status, and bone mineral density.

## Methods

### Study design and study population

**Fig. 1:**
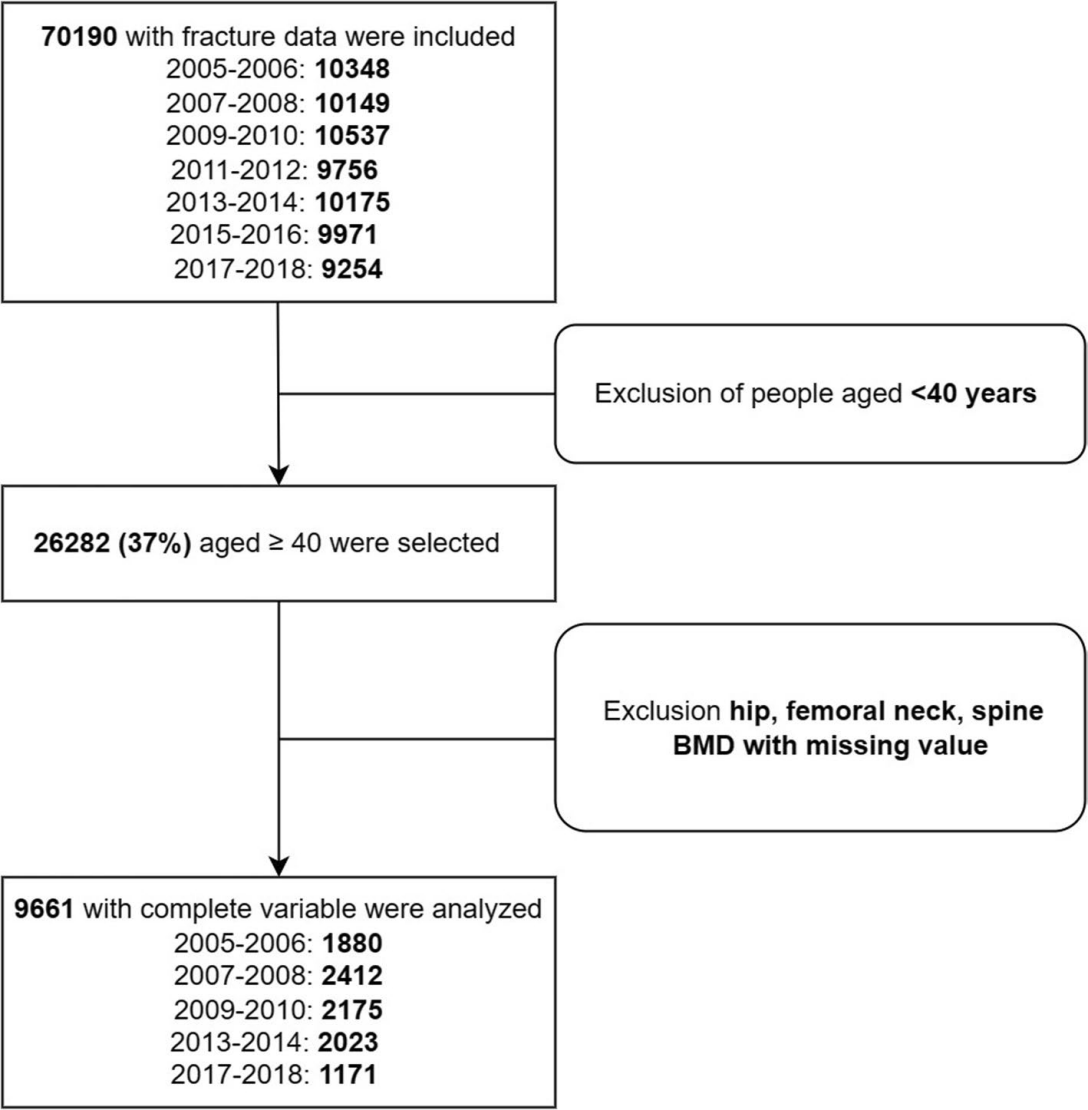
Flow chart of this study

NHANES aimed to collect information about the health and nutrition of families and populations in the United States. It includes Demographic Data, Dietary Data, Examination Data, Laboratory Data, Questionnaire Data, and Limited Access Data. We downloaded the seven cycles (2005–2006; 2007–2008; 2009–2010; 2011–2012; 2013–2014; 2015–2016; 2017–2018) data from the NHANES (Fig. 1**)**. The National Center for health statistics ethical review board approved all NHANES protocols, and written informed consent was obtained from all participants.

### Selection and calculation of weights

As applied in previous studies, for the prediabetes and diabetes definition,the fasting weights was used based on the principle of using the smallest subpopulation weight [13, 14]. However, the fasting weight has a lot of missing values. Therefore, Ten year weights were created by multiplying the 2-year MEC weights by one-fifth. All the variables can be classified into three categories: outcome, exposure, and covariates.

### Outcome variable

Hip BMD, femoral neck BMD, spine BMD were outcome variables. Femur neck and spine BMD was not measured in NHANES in 2011–2012 and 2015–2016. Hence, only five cycles (2005–2006, 2007–2008, 2009–2010, 2013–2014, and 2017–2018) of data were included in the analysis for this study. The spine scans were acquired on Hologic Discovery model A densitometers (Hologic, Inc., Bedford, Massachusetts), using software version Apex 3.2. All scans were analyzed with Hologic APEX version 4.0 software. BMD testing was evaluated by DXA, the examination protocol for which has been described in detail on the NHANES website.

### Definition of diabetes status

The exposure variable was diabetes status. We divided all participants into normoglycemia, pre-diabetes, and diabetes. Impaired Fasting Glycaemia (IFG) and Impaired Glucose Tolerance (IGT) were pre-diabetes. The diagnostic criteria for pre-diabetes are as follows: (1) Participants were told by a doctor that they had pre-diabetes; (2) HbA1c: 5.7–6.5%; (3) FPG: 5.6–7.0 mmol/L; (4) OGTT2: 7.8–11.0 mmol/L.

Diabetes was defined as the following criteria: (1) participants were told by a doctor that they had diabetes; (2) HbA1c > 6.5%;(3) fasting glucose ≥7.0 mmol/L;(4) random blood glucose ≥11.1 mmol/L;(5) two-hour OGTT blood glucose ≥11.1 mmol/L;(6) Use of diabetes medication or insulin. Serum glucose (non-fasting) was measured using a Roche/Hitachi Cobas C Chemistry Analyzer (Roche Diagnostics, Indianapolis, IN) or a Roche/Hitachi Modular P Chemistry Analyzer. HbA1c was measured on a Tosoh Automated Analyzer HLC-723G8 (Tosoh Medics, Inc., San Francisco, CA) or a Tosoh G7 Automated HPLC Analyzer.

### Definition of covariates

Age, Gender, Race, Education, BMI, Smoking, Alcohol user, MET, Protein Intake, Calcium Intake, Vitamin D intake, Serum glucose, HbA1c, Serum Calcium, and Serum Phosphorus, Uric acid were Covariates. Classification standard of BMI: (1) lean: BMI<20 Kg/M^2;(2) normal:BMI ≥ 20 Kg/M^2 and BMI ≤25 Kg/M^2;(3) over: BMI ≥ 25 Kg/M^2. Classification criteria for the degree of smoking. (1) never: smoked less than 100 cigarettes in life; (2) former: smoked more than 100 cigarettes in life and smoke not at all now; (3) now: smoked more than 100 cigarettes in life and smoked some days or every day. The criteria for alcohol consumption are as follows: (1) current heavy alcohol use (≥3 drinks per day for females, ≥4 drinks per day for males, or binge drinking [≥4 drinks on the same occasion for females, ≥5 drinks on the same occasion for males on five or more days per month), (2) current moderate alcohol use (≥2 drinks per day for females, ≥3 drinks per day for males, or binge drinking ≥2 days per month), or (3) a history of daily binge drinking [15].

The metabolic equivalent (MET) was calculated to estimate the average weekly energy expenditure from the Physical Activity Questionnaire. The MET (mins/week) score calculation was available since the 2007–2018 cycle. Take into account that daily dietary intake affects bone density, especially protein and calcium, as well as vitamin D intake. We collected the data from the 2005–2018 cycle.

### Statistical method

We analyzed the data with the R version 4.1.3 software (https://www.R-project.org). If the *P*-value is less than 0.05, the difference is statistically significant. Data are summarized as the Mean (SE) for continuous variables or as a proportion for categorical variables. The linear regression model was used to evaluate the relationship between diabetes status and bone mineral density. We conducted the univariate analysis of outcome variables. We built three linear regression models. In the crude Model, covariates were not adjusted; In Model 2, Age, Gender, and Race were adjusted; In Model 3, all the covariates were adjusted. The subgroup analysis was conducted based on age-group, gender and race.

## Results

### The demographic of participants with different diabetes status

Finally, 9661 participants with complete data were involved in the study. 944 were diagnosed with pre-diabetes (492 participants were IFG, 452 were IGT), and 2043 were with diabetes (Table 1). Compared with the normoglycemia participant, pre-Diabetes and Diabetes patients were older (*P*-value < 0.05). The hip, femoral neck, and spine BMD was higher (P-value < 0.001). The BMI were higher in pre-diabetes and diabetes (P-value < 0.001). Adults who have diabetes had lower MET and education levels (P-value < 0.001). The differences between the two groups were significant in Serum Calcium, Serum Phosphorus, Uric acid, Serum Glucose, and HbA1c (P-value < 0.001).

**Table 1:** Weighted Characteristics of participant with different Diabetes status

Variable	Total (***N*** = 9661)	Normoglycemia (***N*** = 6674)	Pre-Diabetes (***N*** = 944)	Diabetes(***N*** = 2043)	***P***-value
**Year**					**< 0.001**
2005–2006	1880(19.46)	1355(24.048)	191(24.256)	334(19.148)	
2007–2008	2412(24.966)	1596(22.746)	298(29.596)	518(24.692)	
2009–2010	2175(22.513)	1521(20.613)	201(19.463)	453(19.553)	
2013–2014	2023(20.94)	1463(21.696)	164(16.526)	396(19.472)	
2017–2018	1171(12.121)	739(10.898)	90(10.159)	342(17.134)	
**Age**	55.270(0.208)	54.122(0.226)	57.116(0.427)	59.729(0.336)	**< 0.001**
**Gender**					**< 0.001**
Male	4722(48.877)	3145(45.028)	498(53.059)	1079(54.033)	
Female	4939(51.123)	3529(54.972)	446(46.941)	964(45.967)	
**Race**					**< 0.001**
White	4363(45.161)	3224(73.948)	458(72.563)	681(59.266)	
Other	1762(18.238)	1147(10.514)	175(12.167)	440(16.026)	
Black	1918(19.853)	1298(9.534)	139(7.399)	481(14.253)	
Mexican American	1618(16.748)	1005(6.003)	172(7.870)	441(10.455)	
**BMI**					**< 0.001**
Under	427(4.444)	370(5.571)	33(3.312)	24(1.164)	
Over	6826(71.045)	4438(65.283)	713(78.639)	1675(85.066)	
Normal	2355(24.511)	1834(29.147)	193(18.049)	328(13.770)	
**Education**					**< 0.001**
Under High School	2620(27.147)	1574(14.575)	294(20.214)	752(23.687)	
High School or Equivalent	2212(22.92)	1543(23.991)	205(24.754)	464(26.082)	
Above High School	4819(49.933)	3551(61.434)	444(55.032)	824(50.231)	
**Family_Income**					**< 0.001**
Under $50,000	4726(51.136)	3142(38.533)	482(43.789)	1102(45.539)	
Over $50,000	4516(48.864)	3255(61.467)	415(56.211)	846(54.461)	
**Smoke**					**< 0.001**
Never	5110(52.899)	3570(54.482)	465(47.950)	1075(53.599)	
Former	2679(27.733)	1715(25.882)	298(32.463)	666(33.217)	
Now	1871(19.369)	1389(19.635)	181(19.587)	301(13.183)	
**Alcohol Use**					**< 0.001**
Never	1242(14.01)	757(9.004)	141(12.919)	344(16.394)	
Former	1780(20.079)	1104(15.164)	171(15.478)	505(23.560)	
Mild	3257(36.74)	2330(42.033)	327(42.325)	600(38.546)	
Moderate	1270(14.326)	977(18.171)	104(12.856)	189(11.640)	
Heavy	1316(14.845)	976(15.627)	144(16.423)	196(9.861)	
**MET**	3481.478(96.661)	3578.798(110.786)	3176.964(220.643)	3136.710(214.267)	0.068
**Dietary intake**					
Protein intake	81.628(0.666)	81.859(0.706)	82.656(1.687)	79.855(1.498)	0.368
Calcium intake	938.108(10.209)	950.632(11.440)	903.481(24.919)	898.204(16.285)	**0.01**
Vitamin D intake	4.755(0.096)	4.825(0.121)	4.886(0.309)	4.360(0.154)	0.085
**Hematology examination**					
Glucose	5.669(0.026)	5.151(0.011)	5.649(0.025)	8.200(0.112)	**< 0.001**
HbA1c	5.687(0.015)	5.424(0.006)	5.551(0.019)	7.046(0.047)	**< 0.001**
Uric acid	320.380(1.185)	314.179(1.333)	345.290(3.362)	334.731(2.813)	**< 0.001**
Calcium	2.358(0.002)	2.359(0.003)	2.350(0.004)	2.357(0.004)	**0.03**
Phosphorus	1.209(0.003)	1.219(0.003)	1.150(0.008)	1.196(0.007)	**< 0.001**
**Bone mineral density**					
Hip BMD	0.946(0.002)	0.939(0.002)	0.962(0.006)	0.974(0.006)	**< 0.001**
Femoral neck BMD	0.793(0.002)	0.787(0.002)	0.806(0.005)	0.810(0.005)	**< 0.001**
Spine BMD	1.020(0.002)	1.013(0.002)	1.031(0.007)	1.048(0.006)	**< 0.001**

### Univariate analyses of diabetes status and BMD

Univariate analyses were performed between hip BMD, femoral neck BMD, and spine BMD and diabetes status (Table 2). In summary, we found that older, smoking, and drinking groups have lower bone mineral density; women have lower density than men; blacks and Mexican Americans have higher bone density than whites; MET, education level, BMI, family income, protein, calcium, and vitamin D intake were positively correlated with bone mineral density; In the part of Hematology examination, serum glucose, HbA1c, and uric acid were positively correlated with bone density. Blood calcium and phosphorus were negatively correlated with bone mineral density.

**Table 2:** Univariate analysis of BMD and diabetes status

Variable	Hip BMD		Femoral neck BMD		Spine BMD	
	95% CI	***P***-value	95% CI	***P***-value	95% CI	***P***-value
**Age**	−0.005(− 0.005,-0.004)	< 0.001	−0.005(− 0.005,-0.005)	< 0.001	−0.003(− 0.003,-0.002)	< 0.001
**Gender**						
Male	**ref**	**ref**	**ref**	**ref**	**ref**	**ref**
Female	−0.124(− 0.131,-0.117)	< 0.001	− 0.071(− 0.077,− 0.065)	< 0.001	-0.065(− 0.072,-0.058)	< 0.001
**Race**						
White	**ref**	**ref**	**ref**	**ref**	**ref**	**ref**
Other	−0.005(− 0.016,0.006)	0.366	− 0.001(− 0.011,0.008)	0.773	−0.039(− 0.050,-0.028)	< 0.001
Black	0.072(0.062,0.082)	< 0.001	0.093(0.084,0.102)	< 0.001	0.061(0.053, 0.070)	< 0.001
Mexican American	0.038(0.028,0.048)	< 0.001	0.036(0.027,0.045)	< 0.001	− 0.03(− 0.039,-0.021)	< 0.001
**Family income**						
Under $50,000	**ref**	**ref**	**ref**	**ref**	**ref**	**ref**
Over $50,000	0.033(0.025,0.041)	< 0.001	0.023(0.015,0.031)	< 0.001	0.026(0.018,0.034)	< 0.001
**Education**						
Under High School	**ref**	**ref**	**ref**	**ref**	**ref**	**ref**
High School or Equivalent	0.003(−0.009,0.016)	0.579	−0.002(− 0.013,0.010)	0.766	0.022(0.009,0.034)	< 0.001
Above High School	0.013(0.002,0.025)	0.022	0.004(− 0.006,0.014)	0.441	0.031(0.022,0.040)	< 0.001
**BMI**						
Under	**ref**	**ref**	**ref**	**ref**	**ref**	**ref**
Over	0.198(0.184,0.212)	< 0.001	0.143(0.128,0.158)	< 0.001	0.141(0.124,0.157)	< 0.001
Normal	0.084(0.069,0.100)	< 0.001	0.054(0.038,0.071)	< 0.001	0.062(0.044,0.080)	< 0.001
**Smoke**						
Never	**ref**	**ref**	**ref**	**ref**	**ref**	**ref**
Former	0.01(0.002, 0.019)	0.018	−0.002(−0.009,0.006)	0.621	0.009(0.001,0.018)	0.028
Now	− 0.012(− 0.023,− 0.001)	0.039	-0.001(− 0.011,0.008)	0.778	− 0.004(− 0.014,0.006)	0.418
**Alcohol user**						
Never	**ref**	**ref**	**ref**	**ref**	**ref**	**ref**
Former	0.036(0.021,0.052)	< 0.001	0.026(0.013,0.039)	< 0.001	0.034(0.019,0.048)	< 0.001
Mild	0.064(0.048,0.079)	< 0.001	0.042(0.030,0.054)	< 0.001	0.055(0.041,0.069)	< 0.001
Moderate	0.053(0.036,0.070)	< 0.001	0.044(0.030,0.058)	< 0.001	0.049(0.032,0.066)	< 0.001
Heavy	0.091(0.073,0.110)	< 0.001	0.074(0.059,0.089)	< 0.001	0.067(0.052,0.082)	< 0.001
**Diabetes**						
No	**ref**	**ref**	**ref**	**ref**	**ref**	**ref**
Pre-diabetes	0.023(0.011,0.034)	< 0.001	0.018(0.008,0.029)	0.001	0.018(0.004,0.032)	0.010
Diabetes	0.035(0.023,0.046)	< 0.001	0.022(0.013,0.032)	< 0.001	0.035(0.024,0.047)	< 0.001
**MET**	0(0.000,0.000)	< 0.001	0(0.000,0.000)	< 0.001	0(0.000,0.000)	0.036
**Dietary**						
Protein intake	0.001(0.001,0.001)	< 0.001	0.001(0.001,0.001)	< 0.001	0(0.000,0.001)	< 0.001
Calcium intake	0(0.000,0.000)	< 0.001	0(0.000,0.000)	< 0.001	0(0.000,0.000)	< 0.001
Vitamin D intake	0.001(0.000,0.002)	0.008	0.001(0.000,0.002)	0.036	0(0.000,0.001)	0.257
**Hematology examination**						
Glucose	0.006(0.004,0.008)	< 0.001	0.004(0.002,0.005)	< 0.001	0.004(0.003,0.006)	< 0.001
HbA1c	0.011(0.007,0.014)	< 0.001	0.007(0.004,0.010)	< 0.001	0.007(0.004,0.011)	< 0.001
Uric acid	0.001(0.000,0.001)	< 0.001	0(0.000,0.000)	< 0.001	0(0.000,0.000)	< 0.001
Phosphorus	−0.108(−0.133,-0.083)	< 0.001	−0.072(−0.092,-0.051)	< 0.001	−0.095(−0.117,-0.073)	< 0.001
Calcium	−0.112(− 0.159,-0.066)	< 0.001	− 0.088(− 0.127,-0.049)	< 0.001	− 0.13(− 0.175,-0.084)	< 0.001

### Relationship between diabetes status and BMD

We finally built three linear regression models (Table 3). The Crude Model was the non-adjusted model; We adjusted for age, gender, and race in Model 1; All the covariates were adjusted in Model 2.

**Table 3:** Associations Between Diabetes Status and BMD

	Crude model		Model 1		Model 2	
	95% CI	P-value	95% CI	P-value	95% CI	P-value
**Hip BMD**						
No	**ref**		**ref**		**ref**	
Pre-Diabetes	0.023(0.011,0.034)	**0.001**	0.029(0.018,0.039)	< **0.001**	0.016(0.000,0.032)	**0.045**
Diabetes	0.035(0.023,0.046)	< **0.001**	0.048(0.037,0.058)	< **0.001**	0.024(0.007,0.042)	**0.007**
P for trend		< **0.001**		< **0.001**		**0.003**
**Femoral neck BMD**						
No	**ref**		**ref**		**ref**	
Pre-Diabetes	0.018(0.008,0.029)	**0.001**	0.030(0.020,0.040)	< **0.001**	0.022(0.009,0.035)	**0.002**
Diabetes	0.022(0.013,0.032)	< **0.001**	0.041(0.032,0.050)	< **0.001**	0.020(0.004,0.035)	**0.013**
P for trend		< **0.001**		< **0.001**		**0.002**
**Spine BMD**						
No	**ref**		**ref**		**ref**	
Pre-Diabetes	0.018(0.004,0.032)	**0.010**	0.025(0.011,0.039)	< **0.001**	0.023(0.005,0.042)	**0.014**
Diabetes	0.035(0.024,0.047)	< **0.001**	0.048(0.036,0.060)	< **0.001**	0.030(0.011,0.048)	**0.003**
P for trend		< **0.001**		< **0.001**		< **0.001**

Data in the table: OR (95%CI) P-value;

Outcome variable: Hip BMD; Femoral neck BMD; Spine BMD

Exposure variable: Diabetes status； The crude model adjusts for None;

Model I adjusts for Age; Gender; Race;

Model II adjusts for age; gender; race; family_income; edu; bmi_cate; smoke; alcohol.user; PA_total_MET; protein_intake; calcium_intake; vitamin_D_intake; uric_acid;phosphorus; calcium; glucose; HbA1c

In summary, we found that the hip, femoral neck, and spine BMD are positively associated with Diabetes status in the final model (after the adjustment of all the covariates). The specific results wereβ = 0.016, 95% CI:0.000–0.032 for pre-diabetes; β = 0.024, 95% CI: 0.007–0.042 for diabetes in the hip BMD; β = 0.022, 95% CI: 0.009–0.035 for pre-diabetes; β = 0.020, 95% CI: 0.004–0.035 for diabetes in the femoral neck BMD; β = 0.023, 95% CI: 0.005–0.042 for pre-diabetes; β = 0.030, 95% CI: 0.011–0.048 for diabetes in the spine BMD.

### Subgrooup analysis of diabetes status and BMD

Subgroup analysis was carried out based on age group, gender, and race (Table 4). Ultimately, this study found that compared with normoglycemia people, BMD at the hip was lower among white women, women over 50 years of age. We also found the same result for femoral neck BMD. However, for spine BMD, other and black races also had lower BMD.

**Table 4:** The result of subgroup by Age, Gender and Race

Character	Normoglycemia	Pre-diabetes	Diabetes	P for trend	P for interaction
**Hip BMD**					
**Age**					0.43
Under 50	**ref**	0.03(0.01,0.05)	0.04(0.02,0.06)	**< 0.001**	
Over 50	**ref**	0.03(0.02,0.05)	0.06(0.04,0.07)	**< 0.001**	
**Gender**					**0.04**
Male	**ref**	0.01(0.00,0.03)	0.01(0.00,0.02)	0.11	
Female	**ref**	0.01(−0.01,0.03)	0.04(0.02,0.06)	**< 0.001**	
**Race**					0.18
White	**ref**	0.03(0.01,0.04)	0.04(0.02,0.06)	**< 0.001**	
Other	**ref**	0.01(− 0.03,0.05)	0.02(0.00,0.05)	0.08	
Black	**ref**	0(−0.03,0.03)	0.02(− 0.01,0.04)	0.22	
Mexican American	**ref**	0.02(−0.01,0.04)	0(−0.02,0.02)	0.62	
**Femoral neck BMD**					
**Age**					0.88
Under 50	**ref**	0.03(0.01,0.05)	0.04(0.02,0.06)	**< 0.001**	
Over 50	**ref**	0.03(0.01,0.04)	0.04(0.03,0.05)	**< 0.001**	
**Gender**					**0.01**
Male	**ref**	0.01(−0.01,0.02)	0(−0.01,0.02)	0.56	
Female	**ref**	0.02(0.00,0.04)	0.03(0.02,0.05)	**< 0.001**	
**Race**					0.24
White	**ref**	0.03(0.01,0.04)	0.02(0.01,0.04)	**< 0.001**	
Other	**ref**	0(−0.03,0.04)	0.02(0.00,0.04)	0.12	
Black	**ref**	0(−0.03,0.04)	0.01(−0.01,0.03)	0.51	
Mexican American	**ref**	0(−0.02,0.02)	−0.01(− 0.02,0.01)	0.49	
**Spine BMD**					
**Age**					0.08
Under 50	**ref**	0.02(0.00,0.05)	0.03(0.01,0.05)	**0.004**	
Over 50	**ref**	0.02(0.01,0.04)	0.05(0.04,0.07)	**< 0.001**	
**Gender**					0.33
Male	**ref**	0.02(0.00,0.04)	0.03(0.01,0.04)	**< 0.001**	
Female	**ref**	0(−0.02,0.02)	0.03(0.01,0.05)	**0.001**	
**Race**					0.13
White	**ref**	0.03(0.01,0.05)	0.04(0.03,0.06)	**< 0.001**	
Other	**ref**	−0.01(−0.03,0.02)	0.03(0.00,0.06)	**0.03**	
Black	**ref**	0(−0.03,0.03)	0.03(0.00,0.05)	**0.03**	
Mexican American	**ref**	0.01(−0.01,0.04)	0.01(−0.01,0.03)	0.15	

## Discussion

About 422 million people were suffering from abnormal glucose metabolism and depend on insulin to reduce blood glucose levels in their whole life [16]. Patients with diabetes can excrete a large amount of glucose through urine. In addition, a large amount of calcium, phosphorus, and other minerals will also be lost from kidney. If patients do not pay attention to calcium supplements at this time, it is easy to lead to calcium deficiency [17]. Osteoporosis caused by diabetes belongs to secondary osteoporosis. It is a systemic bone disease, resulting in increased bone fragility and fracture prone. The risk of fracture in patients with diabetes combined with osteoporosis is significantly higher than that in healthy people [18].

Our study demonstrated that the bone mineral density of hip, femoral neck, and spine increases in pre-diabetes and diabetes patients, This phenomenon is especially prominent among middle-aged and elderly white women. Many clinical studies have found that the bone mass of diabetic patients can be decreased, increased, or unchanged, but the overall fracture risk is increasing. Our conclusions are consistent with previous studies. Also, Cortet B et al. and Carnevale V et al. have indicated that type 2 diabetes patients are higher BMD than non-diabetes, and the risk of fracture in specific parts was increased [17, 19]. Yan W et al. and Poiana C et al. have proved that T1DM patients had a lower BMD than normoglycemia people; however, T2DM patients had an average or higher BMD [20, 21].

However, at present, many important studies have also confirmed that both pre-diabetic and diabetic patients are more prone to osteoporosis and hip fractures. In general, while bone density increases, so does the risk of osteoporosis and fractures, which appears to be the opposite. We also hope that more and more basic research can explain this problem.

We speculate here that it may be caused by the characteristics of prediabetes and diabetes. Pre-diabetes mainly refers to an intermediate state between normoglycemia and diabetes. It is the necessary stage for the development of diabetes. Glucose metabolism disorders and vitamin D deficiency could change the bone microstructure and matrix [22]. Diabetes damages bone microstructure by inducing abnormal osteocyte function and matrix structure, increasing osteoblast apoptosis, reducing osteoblast differentiation, and enhancing osteoclast-mediated bone resorption [23, 24]. The increased bone density due to obesity does not necessarily provide better protection for bone. However, excessive obesity can make specific body areas more likely to fracture [25, 26].

Pre-diabetes can cause many complications, including kidney problems, vision loss, foot necrosis, and nerve damage. But until now, many diabetes patients and their doctors have not realized it. Hip fractures can be very serious, especially in older women, because they can lead to very high levels of disability. Simunovic N et al. [27] indicated that 20% of people with hip fractures would die within one year of fracture [28]. At the same time, we should eat food rich in vitamin D and exercise scientifically to reduce the risk of hip fracture. Pre-diabetes patients and their caregivers should be aware of the increased risk of hip fractures. Also, Chang W et al. [29] encourage pre-diabetes patients to ask their doctors how to deal with hip fractures.

Bone mineral density in normoglycemia people is closely related to gender, age, and race. There are differences between genders in the same age group, and women are lower than men. The BMD changes with age, and the bone mineral content decreases gradually after 35–40 years old, especially in women. Generally speaking, black people have the highest bone mineral density, white people have the second highest bone mineral density, and Asian people have the lowest bone mineral density [29]. Men’s bone mineral density is higher than women’s [30].

In this study, we have established three models; Model 1 is the crude model; we have adjusted age, gender, and race in model 2, and we adjusted all significant variables of single factor analysis in model 3. Finally, we found that, regardless of age, gender, and race, the BMD of pre-diabetes and diabetes patients is higher than that of normoglycemia people. The results are reliable and stable. At the same time, our study also included other covariates that may affect BMD such as smoking, alcohol drinking, hypertension, physical activity, and dietary protein intake and calcium intake. We have adjusted all the covariates in Model 3 and found that the final results are stable. Similarly, we also take serum calcium, serum phosphorus, serum glucose, and HbA1c. Because HbA1c can reflect the average blood glucose level in the past 8–12 weeks. Blood sugar belongs to the temporary blood sugar status.

The strengths of our study are that first of all, we use a much larger sample size than other retrospective studies, and we used two-years MEC weight in three linear regression models considering that the nhanes was using a complex multi-stage probability sampling method, which makes the conclusions more reliable. However, our study also has limitations. First, the participants suffering from diabetes were defined by the self-reporting of diabetes, Such as age, education level, and smoking were self-reported, which may be inaccurate; Second, a few type 1 diabetic patients may have been included, and the elderly participants with arthritis can skew BMD readings. We can only hope that all the confounding factors should be included.

## Conclusion

Using NHANES data from 2005 to 2018, we found that patients with abnormal glucose metabolism had increased bone mineral density.

## Data Availability

The authors thank the staff and participants of the NHANES study for their valuable contributions. Publicly available datasets were analyzed in this study. This data can be found here: [https://wwwn.cdc.gov/nchs/nhanes/].

## Abbreviations

BMD: Bone mineral density
NHANES: National Health and Nutrition Examination Survey
IFG: Impaired Fasting Glycaemia
IGT: Impaired Glucose Tolerance
MET: Metabolic equivalent
HbA1c: Glycosylated hemoglobin
OR: Odds ratio
Cl: Confidence interval

## References

[1] Middelbeek RJW, Abrahamson MJ (2014). Diabetes, prediabetes, and glycemic control in the United States: challenges and opportunities. Ann Intern Med.

[2] American Diabetes Association. Diagnosis and Classification of Diabetes Mellitus. Diabetes Care. 2013;36(Supplement_1):S67–74. 10.2337/dc13-S067.

[3] Tinajero MG, Malik VS (2021). An update on the epidemiology of type 2 diabetes: a global perspective. Endocrinol Metab Clin N Am.

[4] Kolb H, Martin S (2017). Environmental/lifestyle factors in the pathogenesis and prevention of type 2 diabetes. BMC Med.

[5] Ensrud KE, Crandall CJ (2017). Osteoporosis. Ann Intern Med.

[6] Lane NE (2006). Epidemiology, etiology, and diagnosis of osteoporosis. Am J Obstet Gynecol.

[7] Chau DL, Edelman SV, Chandran M (2003). Osteoporosis and diabetes. Curr Diab Rep.

[8] Sealand R, Razavi C, Adler RA (2013). Diabetes mellitus and osteoporosis. Curr Diab Rep..

[9] Paschou SA, Dede AD, Anagnostis PG, Vryonidou A, Morganstein D, Goulis DG (2017). Type 2 diabetes and osteoporosis: a guide to optimal management. J Clin Endocrinol Metab.

[10] Yao X, Xu X, Jin F, Zhu Z (2020). The correlation of type 2 diabetes status with bone mineral density in middle-aged adults. Diabetes Metab Syndr Obes.

[11] Chen C, Chen Q, Nie B, Zhang H, Zhai H, Zhao L, Xia P, Lu Y, Wang N (2020). Trends in bone mineral density, osteoporosis, and osteopenia among U.S. adults with prediabetes, 2005-2014. Diabetes Care.

[12] Yuan J, Jia P, Zhou JB (2022). Comparison of Bone Mineral Density in US Adults With Diabetes, Prediabetes and Normoglycemia From 2005 to 2018. Front Endocrinol (Lausanne).

[13] Menke A, Casagrande S, Geiss L, Cowie CC (2015). Prevalence of and trends in diabetes among adults in the United States, 1988-2012. JAMA.

[14] Cowie CC, Rust KF, Ford ES, Eberhardt MS, Byrd-Holt DD, Li C, Williams DE, Gregg EW, Bainbridge KE, Saydah SH (2009). Full accounting of diabetes and pre-diabetes in the U.S. population in 1988-1994 and 2005-2006. Diabetes Care.

[15] Rattan P, Penrice DD, Ahn JC, Ferrer A, Patnaik M, Shah VH, Kamath PS, Mangaonkar AA, Simonetto DA (2022). Inverse Association of Telomere Length with Liver Disease and Mortality in the US population. Hepatol Commun.

[16] Chatterjee S, Khunti K, Davies MJ (2017). Type 2 diabetes. Lancet.

[17] Cortet B, Lucas S, Legroux-Gerot I, Penel G, Chauveau C, Paccou J (2019). Bone disorders associated with diabetes mellitus and its treatments. Joint Bone Spine.

[18] Kurra S, Fink DA, Siris ES (2014). Osteoporosis-associated fracture and diabetes. Endocrinol Metab Clin N Am.

[19] Carnevale V, Romagnoli E, D'Erasmo L, D'Erasmo E (2014). Bone damage in type 2 diabetes mellitus. Nutr Metab Cardiovasc Dis.

[20] Yan W, Li X (2013). Impact of diabetes and its treatments on skeletal diseases. Front Med.

[21] Poiana C, Capatina C (2017). Fracture risk assessment in patients with diabetes mellitus. J Clin Densitom.

[22] Sanches CP, Vianna A, Barreto FC (2017). The impact of type 2 diabetes on bone metabolism. Diabetol Metab Syndr.

[23] Brunetti G, D'Amato G, De Santis S, Grano M, Faienza MF (2021). Mechanisms of altered bone remodeling in children with type 1 diabetes. World J Diabetes.

[24] Murray CE, Coleman CM (2019). Impact of Diabetes Mellitus on Bone Health. Int J Mol Sci.

[25] Bonds DE, Larson JC, Schwartz AV, Strotmeyer ES, Robbins J, Rodriguez BL, Johnson KC, Margolis KL (2006). Risk of fracture in women with type 2 diabetes: the Women's Health Initiative observational study. J Clin Endocrinol Metab.

[26] Looker AC, Eberhardt MS, Saydah SH (2016). Diabetes and fracture risk in older U.S. adults. Bone.

[27] Simunovic N, Devereaux PJ, Sprague S, Guyatt GH, Schemitsch E, Debeer J, Bhandari M (2010). Effect of early surgery after hip fracture on mortality and complications: systematic review and meta-analysis. CMAJ.

[28] Nelson DA, Megyesi MS (2004). Sex and ethnic differences in bone architecture. Curr Osteoporos Rep.

[29] Chang W, Lv H, Feng C, Yuwen P, Wei N, Chen W, Zhang Y (2018). Preventable risk factors of mortality after hip fracture surgery: systematic review and meta-analysis. Int J Surg.

[30] Notelovitz M (2002). Overview of bone mineral density in postmenopausal women. J Reprod Med.

